# Ligand Binding Path Sampling Based on Parallel Cascade Selection Molecular Dynamics: LB-PaCS-MD

**DOI:** 10.3390/ma15041490

**Published:** 2022-02-17

**Authors:** Hayato Aida, Yasuteru Shigeta, Ryuhei Harada

**Affiliations:** 1 Graduate School of Science and Technology, University of Tsukuba, 1-1-1 Tennodai, Tsukuba 305-8577, Japan; aida.hayato.sp@alumni.tsukuba.ac.jp; 2 Center for Computational Sciences, University of Tsukuba, 1-1-1 Tennodai, Tsukuba 305-8577, Japan; shigeta@ccs.tsukuba.ac.jp

**Keywords:** PaCS-MD, ligand binding process, ligand–protein complex formation, rare-event sampling method, molecular dynamics simulation, distributed computing

## Abstract

Parallel cascade selection molecular dynamics (PaCS-MD) is a rare-event sampling method that generates transition pathways between a reactant and product. To sample the transition pathways, PaCS-MD repeats short-time MD simulations from important configurations as conformational resampling cycles. In this study, PaCS-MD was extended to sample ligand binding pathways toward a target protein, which is referred to as LB-PaCS-MD. In a ligand-concentrated environment, where multiple ligand copies are randomly arranged around the target protein, LB-PaCS-MD allows for the frequent sampling of ligand binding pathways. To select the important configurations, we specified the center of mass (COM) distance between each ligand and the relevant binding site of the target protein, where snapshots generated by the short-time MD simulations were ranked by their COM distance values. From each cycle, snapshots with smaller COM distance values were selected as the important configurations to be resampled using the short-time MD simulations. By repeating conformational resampling cycles, the COM distance values gradually decreased and converged to constants, meaning that a set of ligand binding pathways toward the target protein was sampled by LB-PaCS-MD. To demonstrate relative efficiency, LB-PaCS-MD was applied to several proteins, and their ligand binding pathways were sampled more frequently than conventional MD simulations.

## 1. Introduction

Self-assembly/organization is a fundamental phenomenon in which well-designed molecules spontaneously gather to form unique structures. These processes are common in nature and are strongly related to molecular recognition, transfer, reaction, and catalysis [1,2,3,4]. Typical examples include self-assembly of a nanocube [5], self-organization of biomolecules such as double helix DNAs and tertiary/quaternary structures of proteins, membrane formation by the self-assembly of lipid bilayers [6], and formation of amyloid fibers [7]. In biological systems, molecular self-assembly/organization plays a crucial role in cell function [8]. Similarly, ligand–protein complex formation upon ligand binding plays an important role in molecular recognition. More specifically, a target protein recognizes a ligand through their ligand–protein interactions. Therefore, from a molecular design point of view, understanding the ligand binding mechanism is essential. Structurally, a target protein complexed with a ligand is determined by various non-covalent interactions, such as hydrogen bonding [9] and van der Waals (vdW) interactions [10]. Finally, a thermodynamically stable complex is selected from possible ligand–protein configurations. However, owing to their complex interactions, the molecular mechanisms of ligand binding processes remain unclear. For this reason, theoretical and computational studies are required to address ligand binding processes at an atomic resolution. In this study, we regard ligand binding processes as self-assembly/self-organization and elucidate their detailed mechanisms using all-atom molecular dynamics (MD) simulations.

As previously described, target proteins recognize their ligands based on ligand–protein interactions. Elucidation of ligand binding pathways is important for understanding the biological functions of complex formations. To form a ligand–protein complex, proteins use their fluctuations to bind to ligands, meaning that protein fluctuation plays a crucial role in ligand recognition. When the target proteins and ligands are rigid, rigid docking simulations predict their ligand–protein complexes. [11,12,13,14] However, it is difficult to treat flexible ligands and/or target proteins with buried binding cavities in terms of rigid docking simulations. Generally, these types of ligand binding processes tend to couple with the conformational transitions of target proteins. To treat ligand binding processes explicitly, MD simulations allow for the evaluation of the flexibility of protein–ligand systems at an atomic resolution. Therefore, MD simulations are increasingly being applied to elucidate ligand–protein binding processes by explicitly considering protein fluctuations.

The characterization of a ligand binding pathway is a challenging issue judging from the timescale gap between conventional MD (cMD) simulations and ligand–protein binding processes. In previous studies, cMD simulations allowed for the sampling of several binding/unbinding events [15,16], some utilizing an Anton (a special-purpose supercomputer) [17,18,19,20]. Notable efforts have also been made in sampling ligand binding pathways using distributed MD simulations combined with Markov state models (MSMs) [21,22] or enhanced sampling methods, such as metadynamics [23,24,25,26,27] and replica exchange methods [28,29,30]. These enhanced sampling methods allow one to sample ligand binding pathways for several proteins.

Generally, ligand binding pathways are regarded as rare events since they are stochastically induced over relatively long timescales, i.e., microseconds, and efficient techniques for sampling a set of ligand binding pathways are yet to be developed. In previous studies, we have developed several rare-event sampling methods based on distributed computing, where their overviews have been introduced in our review papers [31,32]. In distributed computing, short-time MD simulations are independently restarted from important configurations (different initial structures). To promote frequent transitions, a cycle of conformational resampling is repeated, where the cycle consists of (1) selections of the important configurations and (2) the short-time MD simulations from them. When restarting the short-time MD simulations from the different important configurations, their initial velocities are randomly regenerated based on the Maxwell–Boltzmann distribution. As our previous study reported [33], the regenerations of initial velocities promote the conformational transitions of proteins. In restarting the short-time MD, a set of different configurations/velocities works as a perturbation to promote transitions of a target protein. Thus, it is note that LB-PaCS-MD is designated for finding ligand docking pathway and lacks time–series information and exact statistical ensemble. However, once the ligand docking pathways are obtained, the free-energy landscape (FEL) can be accurately evaluated. Here, we evaluated the rough FEL using the conformations sampled by LB-PaCS-MD for the ligand-screening purpose. To increase the transition probability, appropriate initial structures that are relevant to the rare events should be selected by defining reaction coordinates (RCs) that characterize the transitions of the target protein. By referring to the RC values, the initial structures of the short-time MD simulations are appropriately selected. Therefore, the optimal RC should be specified.

As a standard rare-event sampling method, parallel cascade selection MD (PaCS-MD) series [34,35,36,37,38,39] have been proposed. PaCS-MD is categorized into two types: (1) targeted PaCS-MD (t-PaCS-MD) [34] generates transition pathways from a given reactant to a product under the condition that a set of end-point structures (a specified reactant and product) are preliminarily known; (2) non-targeted PaCS-MD (nt-PaCS-MD) [35] generates transition pathways from reactants to neighboring metastable states without referring to any product. t-PaCS-MD and nt-PaCS-MD have been applied to several biological systems to identify rare events and have succeeded in searching broad free-energy landscapes of several proteins [40,41,42].

Based on PaCS-MD, this study concerns the development of ligand binding PaCS-MD (LB-PaCS-MD): a t-PaCS-MD technique to sample ligand binding pathways of a target protein. Under a ligand-concentrated environment, where multiple ligand copies are randomly arranged around a target protein, frequent sampling of the ligand binding pathways is permitted. To select important initial structures of short-time MD simulations, LB-PaCS-MD specifies the center-of-mass (COM) distance between each ligand and the binding site of the target protein, *d*_COM_. By referring to the *d*_COM_ value, snapshots of the short-time MD simulations are ranked; for each cycle, snapshots with smaller *d*_COM_ values are selected as the initial structures. These important configurations are resampled by the short-time MD simulations. With subsequent cycles of LB-PaCS-MD, the *d*_COM_ value gradually decreases and converges to a constant, meaning that a set of ligand binding pathways to the target protein has been successfully sampled by LB-PaCS-MD.

For demonstrations, LB-PaCS-MD was also applied to several ligand–protein complexes, and their ligand binding pathways were successfully sampled with nanosecond-order computational costs. Finally, MSMs were constructed using the sampled ligand binding trajectories to evaluate their free-energy profiles.

## 2. Materials and Methods

### 2.1. T-PaCS-MD

To promote transitions from a given product to a reactant, t-PaCS-MD repeats short-time MD simulations from important configurations. Here, root-mean-square deviation (RMSD) is a simple RC that allows for the selection of configurations that are structurally similar to the product. At every cycle, t-PaCS-MD selects configurations with smaller RMSD values for the product (RMSD_product_) and restarts short-time MD simulations from them. By repeating conformational resampling cycles, the RMSD_product_ value converges to a small constant, indicating that t-PaCS-MD successfully samples the transition pathways from the reactant to product when the following condition is satisfied: RMSD_product_ < cutoff (a threshold). For RCs, any physical variable can be specified. For example, another physical variable has been proposed in our previous study [43].

### 2.2. LB-PaCS-MD

LB-PaCS-MD is an extension of t-PaCS-MD to sample ligand binding pathways toward a ligand–protein complex. In LB-PaCS-MD, a target protein with multiple ligand copies is regarded as a reactant. LB-PaCS-MD samples ligand binding pathways from a ligand-unbounded state to a ligand-bound state. To sample ligand binding pathways frequently, multiple ligand copies were randomly arranged around the target protein. In this ligand-concentrated environment, LB-PaCS-MD more frequently samples ligand binding pathways than a ligand-diluted environment, e.g., the target protein with a single ligand.

**Figure 1:**
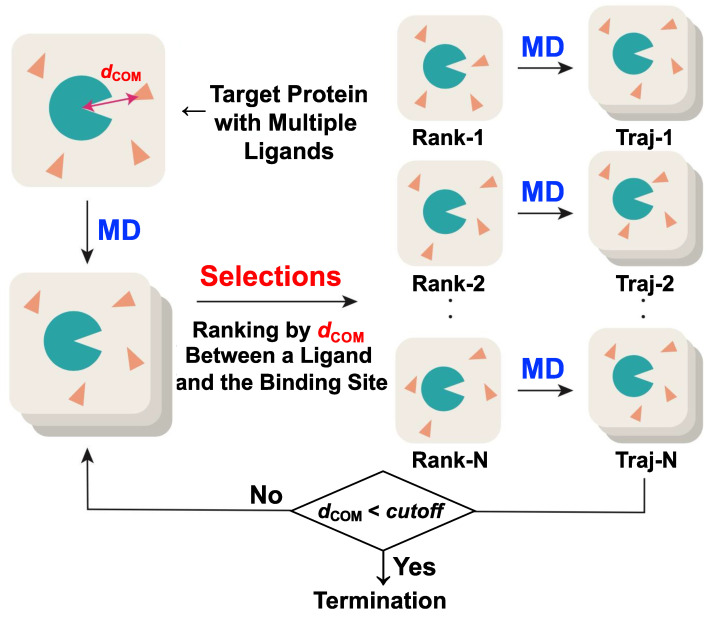
Flowchart of ligand binding path sampling based on LB-PaCS-MD. To frequently sample ligand binding pathways, LB-PaCS-MD repeats conformational resampling cycles consisting of: (1) selections of important configurations (initial structures) with smaller COM distance between the binding site of a target protein and each ligand (*d*_COM_); (2) restarting of (short-time) MD simulations from the selected initial structures. LB-PaCS-MD is terminated when a *d*_COM_ value is less than cutoff (a threshold).

Figure 1 shows the procedure of LB-PaCS-MD. In LB-PaCS-MD, *d*_COM_ is an RC that selects initial structures to be resampled by short-time MD simulations. At every cycle, *d*_COM_ is calculated for all the snapshots generated by the short-time MD simulations at the previous cycle. Here, the total snapshots per cycle is *N* × *M* when *N* short-time MD simulations are independently performed and each short-time MD records *M* snapshots. Then, all the *N* × *M* snapshots are ranked by their *d*_COM_ values, i.e., ascending order in *d*_COM_. Finally, the highly ranked top *N* snapshots with smaller *d*_COM_ values are selected from the *N* × *M* snapshots as initial structures to of *N* short-time MD simulations at the next cycle. The total number of short-time MD simulations (*N*) is specified depending on available computational resources. The large *N* value allows one to sample the ligand binding pathways smoothly. As a strategy to efficiently perform LB-PaCS-MD, it might be better to prevent ligand aggregations during conformational resampling cycles. The ligand aggregations decrease the ligand binding sampling efficiency. Therefore, it is recommended to reset the number of ligands when the arranged ligand copies aggregated during LB-PaCS-MD. By repeating the cycles, the *d*_COM_ value gradually decreases and converges to a constant, indicating that a set of ligand binding pathways toward the binding site of a target protein is sampled by LB-PaCS-MD. To terminate LB-PaCS-MD, one should monitor the *d*_COM_ profiles during the cycles. More specifically, LB-PaCS-MD is terminated when the following condition is satisfied: *d*_COM_ < cutoff (a threshold).

The present strategy for selecting initial structures is applicable for different ligand entries under the condition that more than one ligand exist. In that case, *d*_COM_ is defined as a COM distance between each ligand and each binding site. Therefore, LB-PaCS-MD might be applied to systems with plural ligands as an extension.

### 2.3. Free-Energy Profiles on Ligand-Binding Pathways

To quantitatively evaluate the performance of LB-PaCS-MD, we calculated the free-energy profiles of the sampled ligand-binding pathways using stationary distributions obtained by MSM constructions. The distributions of the ligand-binding trajectories were obtained by projection onto a conformational subspace spanned by reasonable RCs. In this evaluation, *d*_COM_ was selected as an RC. The construction of a reliable MSM consists of two steps: (1) defining microstates and (2) setting a reasonable lag time, *τ*. To define the microstates in step (1), the distributions are clustered using the *k*-means algorithm to yield *N* clusters. Each snapshot is then assigned to the closest cluster (microstate). Next, transitions among the *N* microstates are counted for each trajectory to obtain a maximum likelihood (*N* × *N*) transition matrix, *T*. Each component (*T_ij_*) represents the transition rate from the *i*th to *j*th microstates, with τ averaged over all the trajectories. In step (2), the *i*th slowest implied time, *t_i_*, is estimated from the *i*th largest eigenvalue, λ*_i_*(τ), of *T*, defined as follows:

(1) $$t_{i}\left(\tau \right)=-\frac{\tau }{\ln\lambda_{i}\left(\tau \right)}\;$$

where the optimal value of the implied time is determined by adjusting τ until *t_i_*(τ) reaches an approximately constant value. Convergence of the implied time ensures that the target system satisfies the Markov assumption.

After determining the optimal τ, transitions among the microstates are again counted to estimate *T* under a constrained detailed balance. To calculate the free-energy profile, a stationary distribution, **π** ($\sum_{i}\pi_{i}=1$), is obtained from *T* (**π** = *T* **π**), where **π** corresponds to one of the eigenvectors of *T*. Finally, the free-energy profile under a given temperature (*T*) is calculated for each microstate using the Boltzmann constant (*k*_B_), as follows:

(2) $$F_{i}=-k_{B}T\ln\frac{\pi_{i}}{\underset{k}{\max}\pi_{k}}\;\left(i=1,\;2,\;\ldots \;,\;N\right)$$

where the origin of the free-energy profile is defined by the maximum value of **π**. The following review papers help one to construct MSMs [44,45].

### 2.4. Demonstrations of LB-PaCS-MD

In this study, a set of systems was used to demonstrate LB-PaCS-MD, i.e., LB-PaCS-MD efficiently sampled a set of ligand binding pathways of the following proteins: (1) a mutant of T4 lysozyme (T4L) and (2) SARS-CoV-2 main protease (SARS-CoV2 M^pro^). For T4L, a holo-form was experimentally determined as a protein data bank (PDB) structure, PDBid: 3DMX [46], i.e., T4L complexed with a ligand (benzene molecule). For the SARS-CoV-2 M^pro^, several compounds with binding affinities were screened in previous studies [47,48,49], and four of these compounds were employed in this demonstration. The followings are the simulated compounds, i.e., A—x77; B—methyl rosmarinate; C—niclosamide; D—5,7,3′,4′-tetrahydroxy-2′-(3,3-dimethylallyl. Here, all the cysteine residues of SARS-CoV2 M^pro^ were modelled in their thiole form. For protonation states in both systems, each histidine residue was treated as a histidine with hydrogen on the epsilon nitrogen. To construct a set of parameters of all the compounds, their restrained electrostatic potential (RESP) charges were calculated based on the gas phase HF/6-31G(d) after the structural optimizations based on B3LYP/6-31G(d) as quantum mechanics calculations. Based on the RESP charges, the parameters of all the compound were generated (Supplementary Materials).

**Figure 2:**
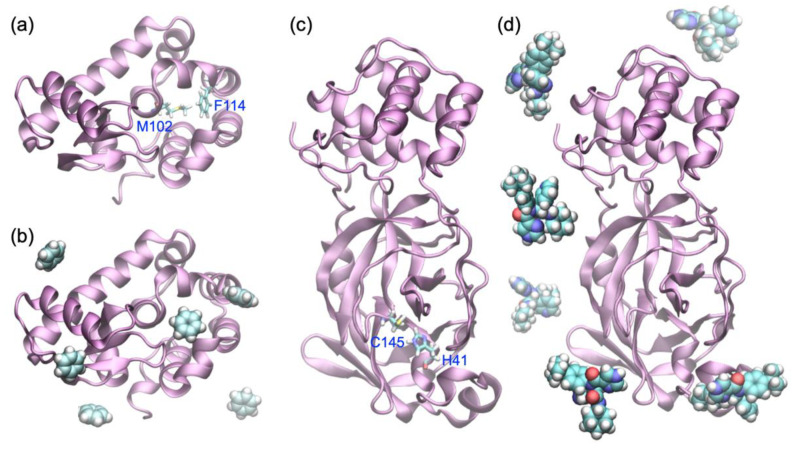
(**a**) Holo-form structure of T4L. The key residues (M102 and F114) around the binding site are depicted in licorice representation. (**b**) T4L with the positions of six randomly arranged ligand copies (benzene molecules in vdW representation). (**c**) Apo-from structure of SARS-CoV2 M^pro^. The dyad residues (H41 and C145) around the binding site are depicted in licorice representation. (**d**) SARS-CoV2 M^pro^ with six randomly arranged ligand copies (compound A molecules in vdW representation).

For both systems, apo-form PDB structures were used as reactants, i.e., 3DMV [46] for T4L and (2) 6M03 for the SARS-CoV-2 M^pro^, without their ligands. Figure 2 shows a set of overviews of both systems. Figure 2a shows the structure of T4L (holo-form), where a set of key residues (M102 and F114) around the binding site is highlighted. Figure 2c shows the structure of SARS-CoV2 M^pro^ (apo form), where a set of catalytic dyad residues (H41 and C145) around the binding site is highlighted.

These structures were solvated with the TIP3P water model [50]. The solvent box sizes were initially set in Å as follows: (*L_x_*, *L_y_*, *L_z_*) = (70.8, 71.0, 71.0) for T4L and (*L_x_*, *L_y_*, *L_z_*) = (87.2, 87.8, 87.6) for SARS-CoV2 M^pro^. To neutralize the solvated systems, counter ions were arranged around each protein. Here, 8 Cl^−^ (4 Na^+^) ions were added to the T4L (SARS-CoV2 M^pro^) system after the solvation. From the modeled systems, 100 ps MD simulations were initiated. Specifically, 100 ps *NVT* (*T* = 300 K) and 100 ps *NPT* (*T* = 300 K and *P* = 1 bar) MD simulations were performed for each solvated configuration. The final snapshots of the 200 ps MD simulations of both systems were regarded as relaxed configurations without their ligands. Before launching each LB-PaCS-MD trial, six ligand copies were randomly arranged around each relaxed configuration and were regarded as the reactants of the LB-PaCS-MD trials. For example, Figure 2b,d show a set of overviews of the positions of the six randomly arranged ligand copies around these proteins. Generally, it is difficult to specify an optimal number of ligand copies. Therefore, we specified the number of ligand copies (*n* = 6) so that the randomly arranged ligand copies in the initial states did not strongly interact with each other to avoid the aggregation.

The following are the MD conditions specified in each LB-PaCS-MD trial. To increase the time step of each MD simulation to 2 fs, the chemical bonds of the solutes were treated with the LINCS algorithm [51]. The chemical bonds of water molecules were treated with the SETTLE algorithm [52]. The modified Berendsen thermostat [53] and Parrinello–Rahman barostat [54,55] were used to control the temperature and pressure, respectively. The particle mesh Ewald method [56] evaluated electrostatic interactions using a real-space cutoff of 10 Å. The cutoff value for the vdW interactions was set to 10 Å. For both demonstrations, all MD simulations were performed using the GPU version of the GROMACS 2018 package [57], where the AMBER 14SBonlysc force field parameter [58] was specified. During the LB-PaCS-MD cycles, all the snapshots were ranked by their *d*_COM_ values. At evert cycle, the highly ranked snapshots were selected as initial structures to be resampled by short-time (100 ps) MD simulations. Therefore, a computational cost per cycle was 1 ns (10 initial structures × 100 ps MD simulations) in both systems.

We first confirmed the validity our MD simulations. Here, to ensure that both proteins stay intact, the averages of backbone RMSD with their standard deviations were calculated using the trajectories of the final LB-PaCS-MD cycles. The averages and standard deviations for each reactant without each ligand were as follows: T4L—1.00 ± 0.14 Å; SARS-CoV2 M^pro^—1.70 ± 0.19 Å (compound A), 1.58 ± 0.27 Å (compound B), 1.56 ± 0.34 Å (compound C), 1.25 ± 0.15 Å (compound D). These backbone RMSD values ensure that both proteins were structurally stable during the LB-PaCS-MD cycles. A set of RMSD profiles versus time of all the systems is given in Figure S1 (Supplementary Materials).

## 3. Results

### 3.1. The Ligand Binding Path Sampling Efficiency of LB-PaCS-MD

The ligand binding path sampling efficiency was analyzed using T4L. As a reactant of LB-PaCS-MD, six benzene molecules were randomly arranged around the apo-form of T4L. Starting from the reactant, three LB-PaCS-MD trials independently sampled a set of ligand binding processes toward the binding site (M102) of T4L. As references, two types of ligand binding searches were considered: (1) three normal PaCS-MD trials under a dilute system (T4L with a single benzene molecule) and (2) a 100 ns cMD simulation under a dilute system (T4L with a single benzene molecule).

To monitor the ligand binding pathways, *d*_COM_ was defined as the COM distance between each benzene molecule and the binding site residue (M102). For T4L, a cutoff value (4.0 Å) was specified based on *d*_COM_ measured in the experimental ligand–protein complex (PDBid: 3DMX). By referring to the PDB structure, each COM was defined for the binding site (M102) and the benzene molecule using their carbon atoms. For the experimental structure, the COM distance between M102 and the benzene molecule was measured as about 4.0 Å. Generally, a cutoff value is specified as a COM distance between each binding site and ligand when some experimental complexes are available. Each COM can be simply defined using coordinates of carbon atoms of each binding site of a target protein and ligand. Note here that this cutoff value was not the firm choice. We have often faced with the problem of deciding when to finish conformational search [59,60,61,62,63]. To be more precise, one may consider the convergence of the distribution evaluated from LB-PaCS-MD. For example, a difference in the distribution of RC values averaged over *n* cycle is one choice since the distribution does not largely change when the conformations reach nearby a product state (a ligand binding structure).

**Figure 3:**
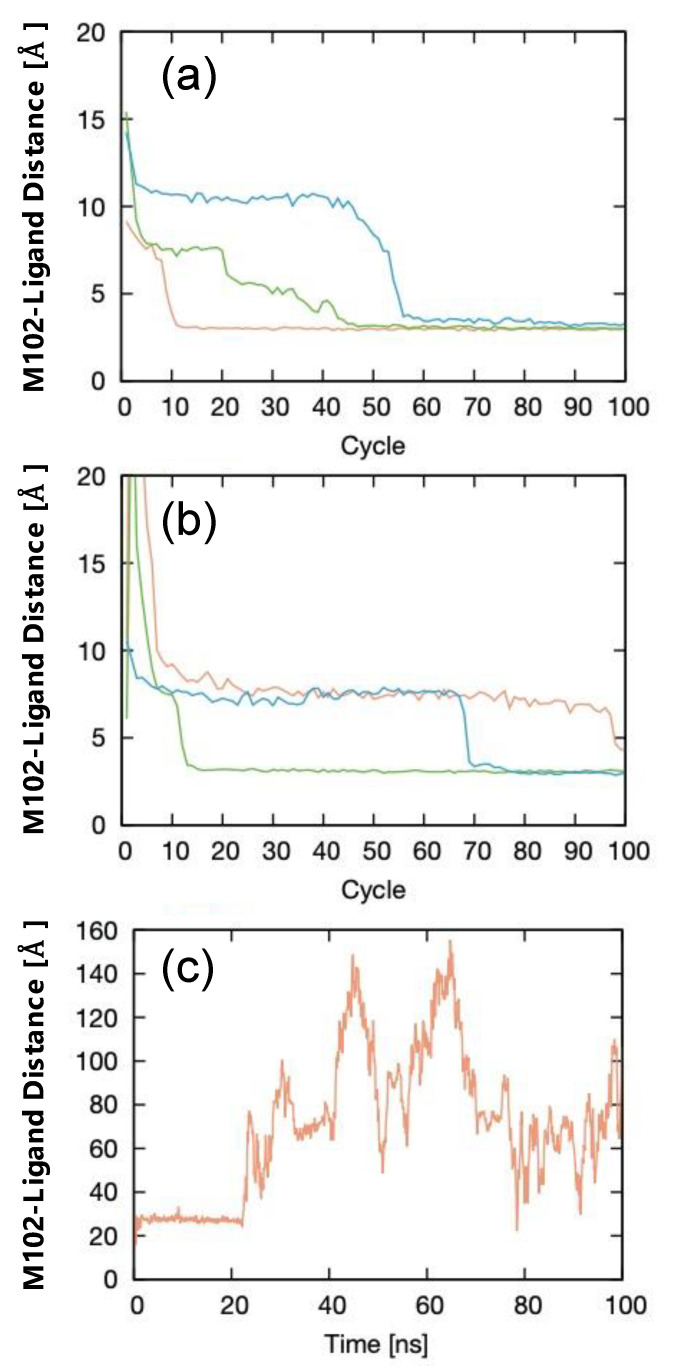
Profiles of the COM distance between each benzene molecule and the binding site residue (M102) of T4L for the ligand-binding trajectories sampled by three LB-PaCS-MD trials. (**a**) LB-PaCS-MD trials using multiple ligand copies. (**b**) Normal PaCS-MD trials using a single ligand. (**c**) The trajectory sampled by a 100 ns cMD simulation under a dilute condition (T4L with one benzene molecule).

Figure 3 shows a set of *d*_COM_ profiles for the three LB-PaCS-MD trials. As shown in Figure 3a, all the LB-PaCS-MD trials successfully sampled the ligand binding pathways until the 100th cycle (100 ns), i.e., all the *d*_COM_ profiles converged to small values (*d*_COM_ ~ 3.7 Å) after the 100th cycle. Here, 1 of the 3 normal PaCS-MD trials failed to sample the ligand-binding pathways after the 100th cycle (*d*_COM_ ~ 4.7 Å). In contrast, the 100 ns cMD simulations failed to sample pathways at the same computational cost, indicating a higher sampling efficiency of LB-PaCS-MD than both normal PaCS-MD and cMD.

Here, the ligand binding forms sampled by LB-PaCS-MD were compared with the X-ray structure. Figure S2a (Supplementary Materials) shows a sampled ligand binding snapshot, where it was superimposed for the X-ray structure. In Figure S2a, the key residue at the binding site (M102) is depicted in vdW representation, and each benzene molecule is depicted in licorice representation (blue—LB-PaCS-MD; red—the X-ray structure). The RMSD value between these benzene molecules after superimposition of T4L was 0.79 Å. To focus on the ligand binding form, a set of key residues (M102 and F114) around the binding site was depicted with each ligand (Figure S2b,c). In both binding forms, the benzene molecules maintained the common interaction with M102. For the binding form sampled by LB-PaCS-MD (Figure S2b, Supplementary Materials), the ligand had an additional contact with F114, which might be a weak stacking of their aromatic rings. As previous studies reported [64,65], the binding cavity of T4L tends to fluctuate to catch the ligand since the key residues around the binding site is deeply buried and sterically inaccessible from bulk solvent, indicating that the binding form of the ligand might be affected by the thermal fluctuation of the binding cavity and takes variations.

### 3.2. Elucidations of the Ligand Binding Pathways of SARS-CoV2 Main^Pro^

For the second demonstration, LB-PaCS-MD was applied to sample a set of ligand- binding pathways of SARS-CoV-2 M^pro^. Based on a previous study that screened compounds with binding affinities [47,48,49], four compounds were used as a set of ligand candidates. To model each complex system, six ligand copes were randomly arranged around SARS-CoV-2 M^pro^. For SARS-CoV-2 M^pro^, the complex structures have not been experimentally determined, i.e., the explicit *d*_COM_ values are unavailable. Therefore, a common cutoff value (6.0 Å) was used to terminate the LB-PaCS-MD cycles. Starting from each reactant, the LB-PaCS-MD trials successfully sampled the ligand binding pathways of SARS-CoV-2 M^pro^.

**Figure 4:**
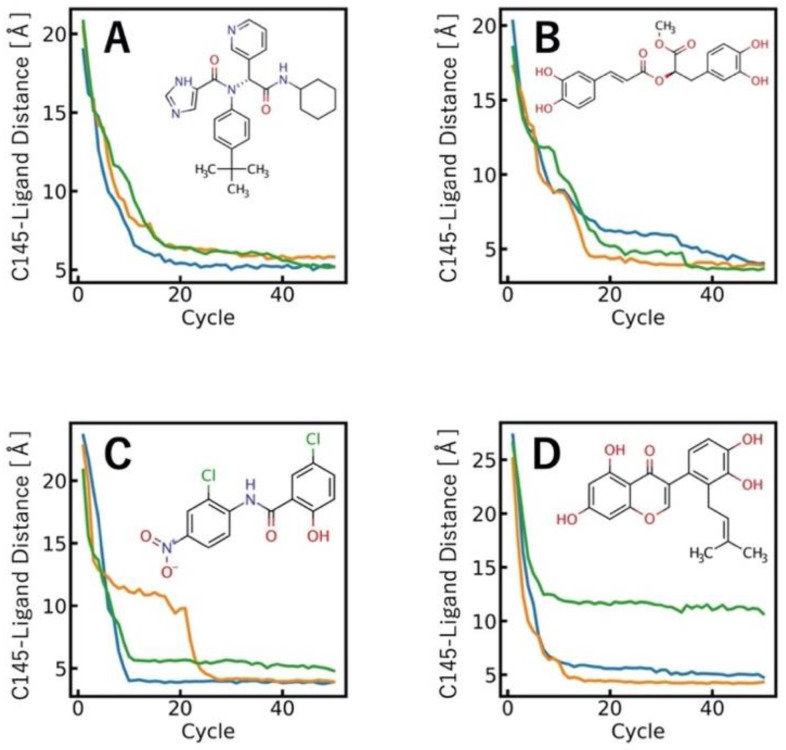
Profiles of the COM distances between the binding site residue (C145) of SARS-CoV-2 M^pro^ and all the compounds for their ligand binding trajectories. (**A**) x77; (**B**) methyl rosmarinate; (**C**) niclosamide; (**D**) 5,7,3′,4′-tetrahydroxy-2′-(3,3-dimethylallyl) isoflavone.

Figure 4 shows a set of *d*_COM_ profiles between each compound and the binding site residue (C145). The *d*_COM_ values converged sufficiently until the 50th cycle. All the LB-PaCS-MD trials successfully sampled the ligand binding pathways for compounds A, B, and C. In contrast, one of the three LB-PaCS-MD trials failed to sample the ligand-binding pathways for compound D. Based on these analyses, LB-PaCS-MD allows one to sample a set of ligand-binding pathways of a given target protein with a reasonable (nanosecond-order) computational cost.

**Figure 5:**
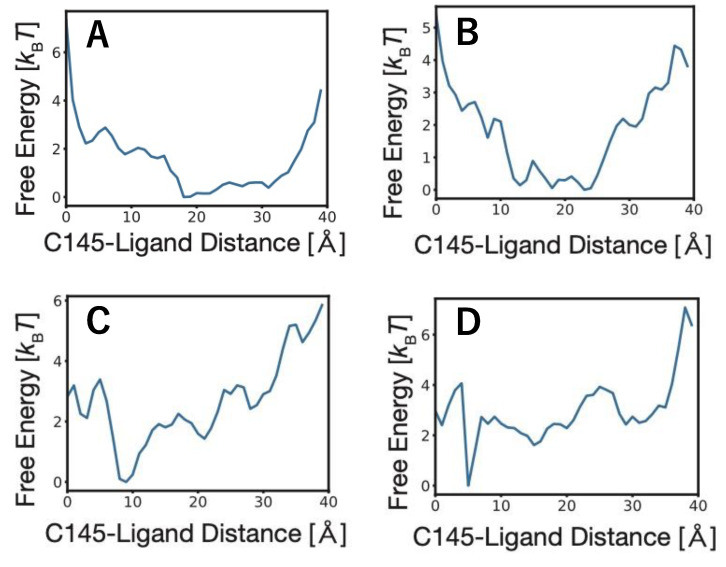
Free-energy profiles projected onto *d*_COM_ of the ligand binding pathways sampled by three LB-PaCS-MD trials for compounds A, B, C, and D. *d*_COM_ is defined as the COM distance between the binding site residue (C145) and each compound. (**A**) x77; (**B**) methyl rosmarinate; (**C**) niclosamide; (**D**) 5,7,3′,4′-tetrahydroxy-2′-(3,3-dimethylallyl) isoflavone.

The ligand binding pathways sampled by the LB-PaCS-MD trials were evaluated quantitatively by constructing MSMs. The MSMs were constructed using the distributions projected onto the *d*_COM_ subspace, providing a set of stationary distributions that allowed for the calculation of free-energy profiles as a function of *d*_COM_ (Figure 5). The free-energy profiles showed characteristic covertures along *d*_COM_. Especially, the free-energy profiles for compounds C and D had steep valleys within 10 Å of *d*_COM_, while those for compounds A and B had gradual valleys around 20 Å of *d*_COM_, indicating that compounds C and D had higher binding affinities than compounds A and B. As a relative comparison of the binding affinities between compounds B and D, a previous study evaluated their values [48]. More specifically, the binding affinity of compound D (−29.57 kcal/mol) was higher than that of compound B (−20.62 kcal/mol). Here, compound D showed the highest binding affinity in the considered compounds [48], which reflects the covertures of the free-energy profiles of these compounds, validating compound D with the higher binding affinity had the steep valley along *d*_COM_, while compound B with the lower binding affinity had the gradual valley along *d*_COM_.

**Figure 6:**
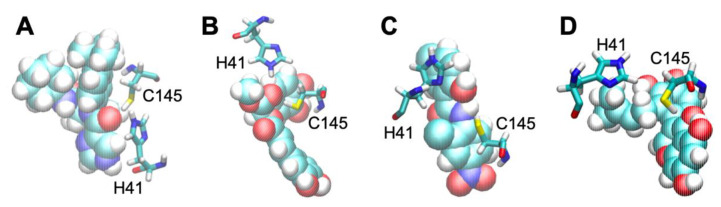
Ligand–protein interactions for compounds (**A**–**D**). Each compound is depicted in vdW representation. The dyad residues (H41 and C145) around the binding site are depicted in licorice representation.

To address the interaction between the binding site and each compound, the characteristic snapshots sampled at the final (50th) cycle are shown in Figure 6. To highlight the ligand–protein interactions, the catalytic dyad residues (C145 and H41) around the binding site were depicted. The importance of these residues has been already reported [48]. Judging from Figure 6, all the compounds interacted with the thiole and imidazole groups of C145 and H41. As shown in Figure 6, compounds A and B interacted with these groups via a single site. In contrast, compounds C and D interacted with these groups via multiple sites. The difference in the interaction patterns might reflect the difference in the binding affinities of these compounds. More specifically, compound A and B tend to fluctuate due to the single site interaction, which might be an origin of the gradual valley in the free-energy profiles. Similarly, compounds C and D tend to tightly interact with the catalytic dyad residues via the multiple sites, which might be an origin of the steep valley in the free-energy profiles.

In the present study, one chain of SARS-CoV2 M^pro^ was used to perform the short-time MD simulations. As a previous study reported [66], the protein dynamics and binding pocket geometry might be different between monomer and dimer. However, we used the monomer to sample the ligand binding paths to the monomer as a simple demonstration since the dimer simulation becomes complicated and takes longer time. When simulating the dimer, LB-PaCS-MD must consider the double binding sites. Since one of us has already performed the dimer simulation with a ligand in the A chain [67,68], the dimer simulation will be a next application of LB-PaCS-MD for the purpose of in silico screening of compounds.

In addition, the simple protonation for the HIS residues might not reflect the physiological state rigorously. Owing to the present system setup, the SARS-CoV2 M^Pro^ monomer with the simple HIS protonation was only used as a demonstration. Therefore, it might be difficult to derive biological/pharmaceutical implications from the preset results.

## 4. Discussion

The ligand binding path sampling efficiency of LB-PaCS-MD might depend on the number of ligand copies to be arranged around a target protein. Generally, it is difficult to optimally arrange multiple ligand copies, meaning that the optimal number is unknown. Another concern was that the arranged ligand copies might aggregate at high concentrations, which would result in insufficient ligand binding path sampling. For this issue, weakly repulsive intermolecular interactions among the arranged ligand copies might prevent aggregation. These highly ligand-concentrated environments with the repulsive intermolecular interactions enhance ligand binding site searches on protein surfaces [69].

For an RC of LB-PaCS-MD, the COM distance between a ligand and the binding site of a target protein was specified as an RC. However, future studies should consider other RCs to characterize more complicated binding poses. In addition to *d*_COM_, some RCs might be more appropriate for characterizing ligand orientations upon binding to the active site. To treat complicated ligand binding pathways, a scoring function could help to select important configurations and can be defined by multiple RCs. Ligand binding path sampling in a higher-dimensional RC subspace based on the scoring function could theoretically increase the ligand path-sampling efficiency of LB-PaCS-MD.

Here, we discuss the validity of *d*_COM_ as a measure when COM of a ligand is flexible. To treat the flexibility, the shape of a target ligand is characterized by its radius of gyration (*R*_g_) or accessible surface area (SASA). These measures allow one to treat a flexible ligand rather than a rigid one. To select initial structures during LB-PaCS-MD cycles, a scoring function can be defined using *R*_g_ and SASA in addition to *d*_COM_. To treat this delicate ligand binding process, both *d*_COM_ and *R*_g_ might be appropriate measures to rank snapshots by considering the ligand shape. The definition of a reasonable scoring function with multiple measures might be important to sample more complicated ligand-binding pathways. Figure S3 (Supplementary Materials) shows a comparison to explain a difference in the ligand-binding path sampling strategy using a single RC and multiple RCs in LB-PaCS-MD.

To quantitatively calculate the binding and dissociation constants of a ligand for a target protein, intensive sampling of both binding and unbinding pathways is required. For the dissociation path sampling, we have proposed several methods based on distributing computing [31,32]. Therefore, combinations of these dissociation sampling methods with LB-PaCS-MD allow one to intensively sample both biding and unbinding trajectories, enabling one to estimate the binding and dissociation constants quantitatively.

Instead of *d*_COM_, an alternative RC might be considered to characterize intermolecular interactions between a target protein and a ligand. For example, the intermolecular interactions might be described by a distance matrix, whose matrix elements correspond to atom-to-atom distances between the ligand and protein. To select important configurations in LB-PaCS-MD, the difference between a pair of distance matrices of an instantaneous snapshot ($A_{ij}$, the distance between the *i*th and *j*th atoms), and the target complex ($B_{ij}$, the distance between the *i*th and *j*th atoms) is regarded as a reasonable measure. As an option, these distance matrices can be locally defined for atoms around the binding site not all the atoms of the target protein complex. For example, a sum of all the elements of the difference matrix ($\Delta =\sum_{ij}A_{ij}-B_{ij}$) can be defined as a measure. In terms of Δ, LB-PaCS-MD selects snapshots with smaller Δ values as important configurations by considering the ligand–protein intermolecular interactions. According to cycles, the Δ value gradually decreases as the ligand binding process proceeds.

Judging from ligand dynamics, its diffusion might play an important role in binding to a target protein. The diffusion of the ligand is described by the mean-square-deviation (MSD) of COM of a target ligand. To consider the mobility, MSD of the ligand might be a measure in addition to *d*_COM_ to define a reasonable scoring function for the selections of important configurations in LB-PaCS-MD.

Next, we discuss an extension of LB-PaCS-MD for sampling other self-assembly processes. In this study, we only treated ligand–protein complex formation using T4L and SARS-CoV-2 M^pro^. In addition to these biological targets, LB-PaCS-MD might be applicable to the self-assembly of materials such as nanocubes of organic compounds. In a previous study, a set of dissociation processes of a carbon nanocube (hexamer) was sampled using our proposed rare-event sampling method [5,70,71]. In the sampled dissociation processes, the hexamer was decomposed into monomers. As a post-processing treatment after sampling the dissociation processes based on other rare-event sampling methods [72,73], LB-PaCS-MD might inversely sample a set of association processes from the dissociated monomers toward the self-assembled hexamer. Therefore, LB-PaCS-MD might allow one to sample a set of self-assembly processes of organic molecules toward a higher-order structure. In summary, LB-PaCS-MD can be used to elucidate several self-assembly processes, not only biological molecules, but also organic molecules.

Finally, we compare LB-PaCS-MD with other enhanced ligand sampling methods for their ligand-binding path sampling strategy. In a review paper [74], several enhanced ligand binding path sampling methods have been introduced. About the sampling of ligand binding pathways, umbrella sampling [75,76,77] or metadynamics and their variants [78,79,80,81] adopted external biases along a specified RC such as *d*_COM_. In these biased sampling methods, RCs are specified prior to applications. In this sense, LB-PaCS-MD have a common issue of RC specifications with these biased sampling methods. For implementation, LB-PaCS-MD can be performed using a simple script, allowing one to control the conformational resampling cycles without modifying source codes and imposing external biases. For a disadvantage, LB-PaCS-MD is difficult to be applied when the ligand binding site of a target protein is unknown. Therefore, alternative methods are required to identify the binding sites prior to applications. For alternative methods, the cosolvent or mixed-solvent MD simulations are regarded as computational tools in identifying the ligand binding sites [82,83]. These methods use solvent mixtures that usually consist of organic molecules or fragments that possessed key pharmacophore features crucial for ligand binding. Once the cosolvent or mixed solvent MD simulations identified the ligand binding sites, LB-PaCS-MD intensively samples the ligand binding paths toward the hotspots.

## 5. Conclusions

In this study, LB-PaCS-MD, an extension of PaCS-MD, was used to frequently sample ligand binding pathways toward a target protein. Multiple ligand copies were randomly arranged around a given target protein to increase the chance of sampling ligand-binding pathways. In a ligand-concentrated environment, LB-PaCS-MD allowed for frequent ligand binding path sampling. LB-PaCS-MD specified the COM distances between each ligand and the binding site residue of the target protein, *d*_COM_. The snapshots generated by LB-PaCS-MD were ranked by their *d*_COM_ values. In each cycle, snapshots with smaller *d*_COM_ values were selected as important configurations (initial structures), and they were conformationally resampled by short-time MD simulations. By repeating conformational resampling cycles, the *d*_COM_ value gradually decreased and converged to a constant, indicating that a set of ligand binding pathways was successfully sampled by LB-PaCS-MD. As demonstrations, LB-PaCS-MD was applied to two proteins (T4L and SARS-CoV-2 M^pro^), and their ligand binding pathways were sampled more frequently than both normal PaCS-MD and cMD in the dilute environments, validating the higher performance of ligand-binding path sampling.

In distributed computing, such as LB-PaCS-MD, it is essential to select important configurations with high potential to make transitions toward a target state. The important configurations are selected based on a criterion for defining the high potential of snapshots. Generally, the criterion is non-trivial and empirically determined depending on the target system. Therefore, several measures (physical variables) are considered to define the criterion. For a future perspective, these measures might be automatically determined by a machine learning algorithm. For example, our previous study [84] adopted a machine learning like algorithm to automatically search a set of optimal measures for selecting the important configurations. When selecting the important configurations in LB-PaCS-MD, some machine learning algorithms might increase the ligand-binding sampling efficiency.

## Data Availability

No new data were created or analyzed in this study. Data sharing is not applicable to this article.

## Supplementary Materials

The following supporting information can be downloaded at: https://www.mdpi.com/article/10.3390/ma15041490/s1, 1. Supplementary Materials for the T4L and SARS-CoV2 M^pro^ Systems: Figure S1: Time series of the backbone RMSD of each protein. For each system, the trajectories generated by each final cycle (the 100th cycle for T4L and the 50th cycle for SARS-CoV2 M^pro^) were used to calculate the backbone RMSD values. For the RMSD calculations, each relaxed configuration (the snapshots after the 200-ps MD simulations from the experimental structures for both systems) was used as each reference. (a) T4L. (b) SARS-CoV2 M^pro^ with compound A. (c) SARS-CoV2 M^pro^ compound B. (d) SARS-CoV2 M^pro^ compound C. (e) SARS-CoV2 M^pro^ compound D.; Figure S2: (a) Ligand-binding form sampled by LB-PaCS-MD. The key residue (M102) for the ligand-binding process is depicted in vdW representation with each benzene molecule in licorice representation (blue: LB-PaCS-MD, red: the X-ray structure). (b, c) The ligand-binding forms are highlighted around the binding site with a set of key residues (M102 and F114). (top) The benzene molecule sampled by LB-PaCS-MD. (bottom) The benzene molecule in the X-ray structure.; Figure S3: Flowchart of selections of initial configurations in LB-PaCS-MD. (a) Selections of initial configurations using a single reaction coordinate (RC) and (b) those using multiple RCs in terms of a pre-defined scoring function. 2. Parameter Files for All the Organic Compounds (Benzene, Compound A, B, C, and D).
