# Supplementary material for: Ligand Binding Path Sampling Based on Parallel Cascade Selection Molecular Dynamics: LB-PaCS-MD
Source: Materials (Basel). 2022 Feb 17;15(4):1490. doi: 10.3390/ma15041490 (PMC8878848; doi:10.3390/ma15041490)
Supplement: Supplementary file 1 [file materials-15-01490-s001.zip › materials-1545416-supplementary.pdf]

materials

Supplementary Material

Ligand Binding Path Sampling Based on Parallel Cascade Selection Molecular Dynamics: LB-PaCS-MD

Hayato Aida [1], Yasuteru Shigeta [2] and Ryuhei Harada [2,*]

[1] Graduate School of Science and Technology, University of Tsukuba, 1-1-1 Tennodai, Tsukuba 305-8577, Japan; aida.hayato.sp@alumni.tsukuba.ac.jp
[2] Center for Computational Sciences, University of Tsukuba, 1-1-1 Tennodai, Tsukuba 305-8577, Japan; shigeta@ccs.tsukuba.ac.jp
* Correspondence: ryuhei@ccs.tsukuba.ac.jp

1. Supplementary Materials for the T4L and SARS-CoV2 M[pro] Systems

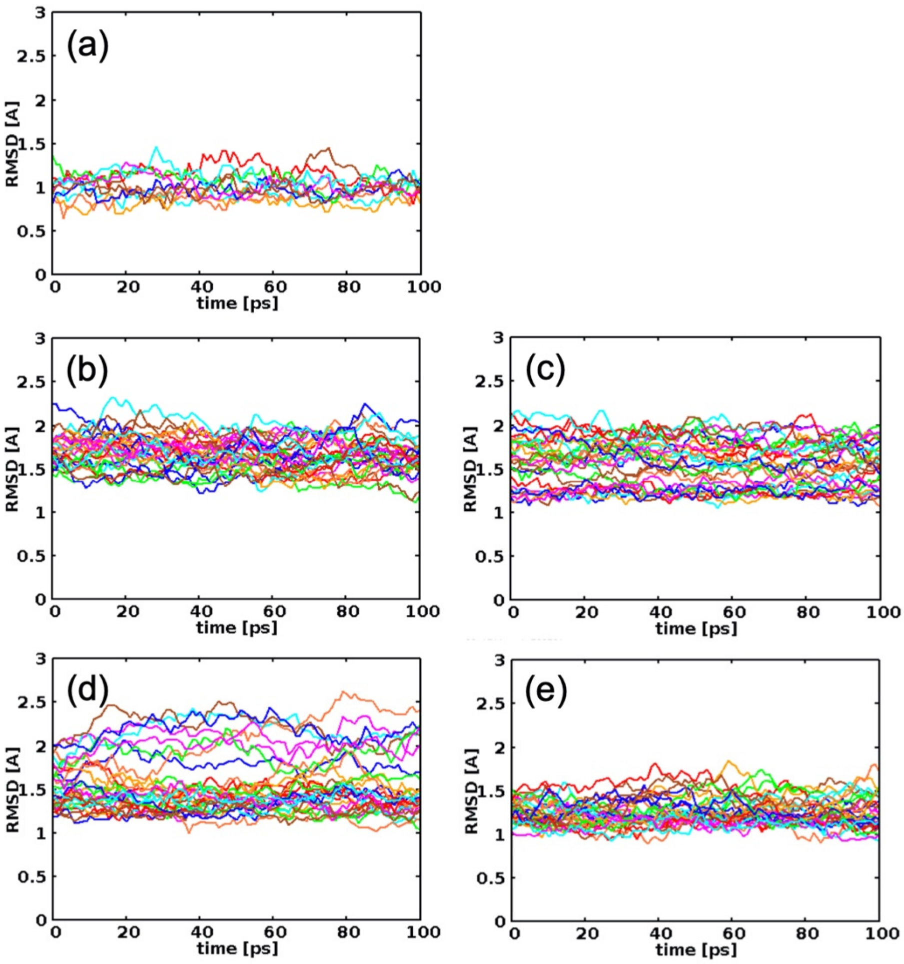

Figure S1. Time series of the backbone RMSD of each protein. For each system, the trajectories generated by each final cycle (the 100th cycle for T4L and the 50th cycle for SARS-CoV2 M[pro]) were used to calculate the backbone RMSD values. For the RMSD calculations, each relaxed configuration (the snapshots after the 200-ps MD simulations from the experimental structures for both systems) was used as each reference. (**a**) T4L. (**b**) SARS-CoV2 M[pro] with compound A. (**c**) SARS-CoV2 M[pro] compound B. (**d**) SARS-CoV2 M[pro] compound C. (**e**) SARS-CoV2 M[pro] compound D.

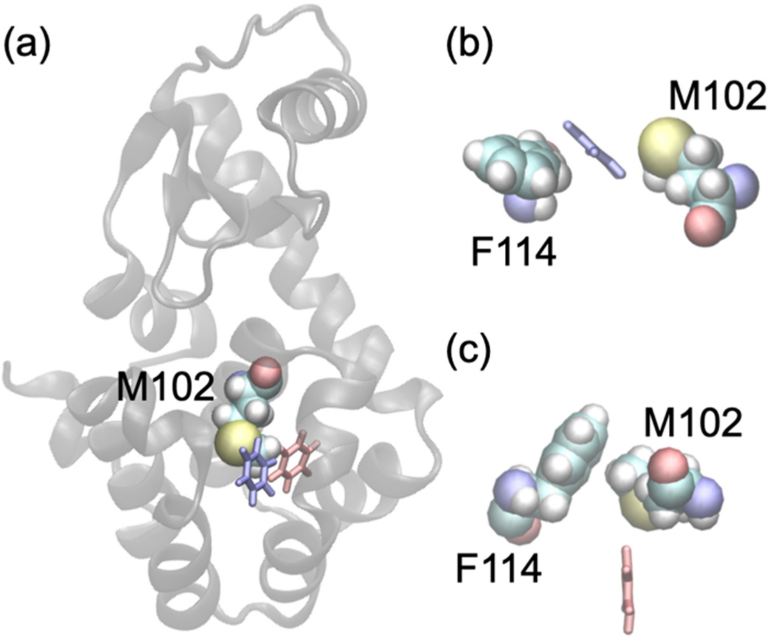

Figure S2. (**a**) Ligand-binding form sampled by LB-PaCS-MD. The key residue (M102) for the ligand-binding process is depicted in vdW representation with each benzene molecule in licorice representation (blue: LB-PaCS-MD, red: the X-ray structure). (**b**,**c**) The ligand-binding forms are highlighted around the binding site with a set of key residues (M102 and F114). (top) The benzene molecule sampled by LB-PaCS-MD. (bottom) The benzene molecule in the X-ray structure.

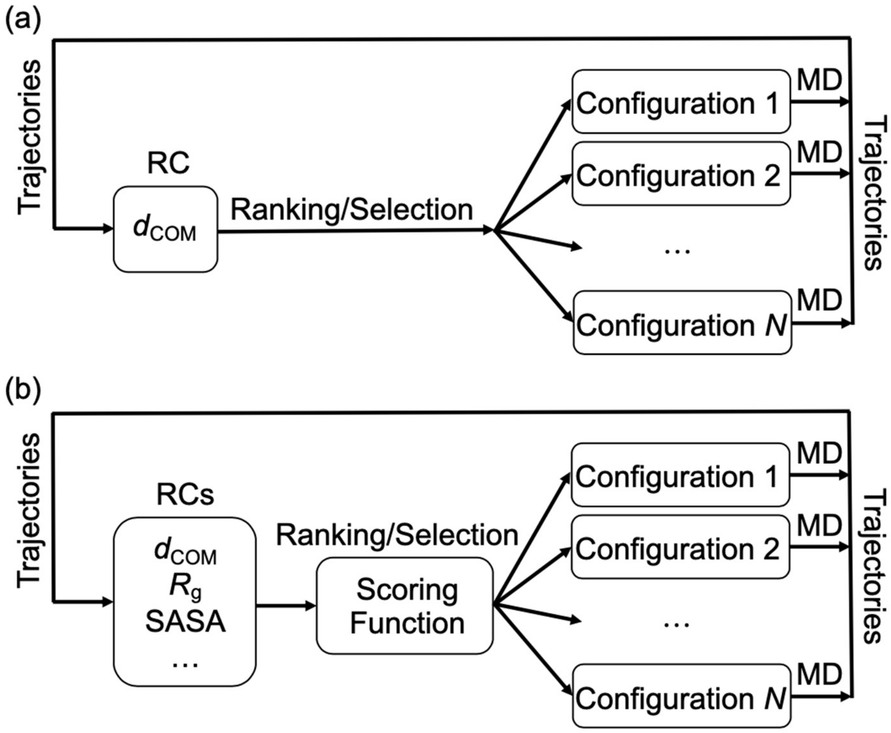

Figure S3. Flowchart of selections of initial configurations in LB-PaCS-MD. (**a**) Selections of initial configurations using a single reaction coordinate (RC) and (**b**) those using multiple RCs in terms of a pre-defined scoring function.

2. Parameter Files for All the Organic Compounds (Benzene, Compound A, B, C, and D)

The followings are the amber prep and frcmod files to perform MD simulations of the organic compounds.

benzene.frcmod

remark goes here
MASS

BOND

ANGLE

DIHE

IMPROPER
ca-ca-ca-ha 1.1 180.0 2.0 General improper torsional angle (2 general atom types)

NONBON

benzene.prep

```
   0    0    2

This is a remark line
molecule.res
BNZ    INT   0
CORRECT      OMIT DU    BEG
  0.0000
   1  DUMM   DU     M    0   -1   -2      0.000      .0        .0       .00000
   2  DUMM   DU     M    1    0   -1      1.449      .0        .0       .00000
   3  DUMM   DU     M    2    1    0      1.523   111.21       .0       .00000
   4  C1      ca    M    3    2    1      1.540   111.208  -180.000 -0.120310
   5  H1      ha    E    4    3    2      1.070    24.218  -179.739  0.120310
   6  C2      ca    M    4    3    2      1.397   144.319   179.930 -0.120310
   7  H2      ha    E    6    4    3      1.071   119.784    -0.104  0.120310
   8  C3      ca    M    6    4    3      1.396   120.398   179.890 -0.120310
   9  H3      ha    E    8    6    4      1.070   120.087  -179.580  0.120310
  10  C4      ca    M    8    6    4      1.397   119.880     0.427 -0.120310
  11  H4      ha    E   10    8    6      1.070   120.129   179.364  0.120310
  12  C5      ca    M   10    8    6      1.395   119.643    -0.660 -0.120310
  13  H5      ha    E   12   10    8      1.071   119.762  -179.333  0.120310
  14  C6      ca    M   12   10    8      1.397   120.544     0.712 -0.120310
  15  H6      ha    E   14   12   10      1.070   120.127   179.464  0.120310


LOOP
   C6    C1

IMPROPER
   C6    C2    C1    H1
   C1    C3    C2    H2
   C2    C4    C3    H3
   C5    C3    C4    H4
   C6    C4    C5    H5
   C5    C1    C6    H6

DONE
STOP
```

A.frcmod

remark goes here
MASS

BOND

ANGLE

DIHE

IMPROPER
ca-ca-ca-ha 1.1 180.0 2.0 General improper torsional angle (2 general atom types)
ca-ca-ca-n 1.1 180.0 2.0 Using default value
c -c3-n -ca 1.1 180.0 2.0 Using default value
cc-n -c -o 10.5 180.0 2.0 General improper torsional angle (2 general atom types)
c -cd-cc-nc 1.1 180.0 2.0 Using default value
cc-h4-cd-na 1.1 180.0 2.0 Using default value
cd-cd-na-hn 1.1 180.0 2.0 General improper torsional angle (2 general atom types)
h5-na-cd-nc 1.1 180.0 2.0 Using default value
ca-h4-ca-nb 1.1 180.0 2.0 Using default value
c3-n -c -o 10.5 180.0 2.0 General improper torsional angle (2 general atom types)

NONBON

A.prep

```
    0    0    2

This is a remark line
molecule.res
LIG    INT   0
CORRECT        OMIT DU    BEG
  0.0000
   1  DUMM   DU     M    0  -1  -2     0.000      .0        .0      .00000
   2  DUMM   DU     M    1   0  -1     1.449      .0        .0      .00000
   3  DUMM   DU     M    2   1   0     1.523   111.21       .0      .00000
   4  C20    c3     M    3   2   1     1.540   111.208  -180.000 -0.442270
   5  H19    hc     E    4   3   2     1.096   103.783   176.498  0.096260
   6  H20    hc     E    4   3   2     1.097   146.340    15.454  0.096260
   7  H21    hc     E    4   3   2     1.095    50.892    74.721  0.096260
   8  C19    c3     M    4   3   2     1.548    66.546   -76.394  0.592550
   9  C21    c3     3    8   4   3     1.540   108.114  -158.610 -0.442270
  10  H22    hc     E    9   8   4     1.096   111.825  -178.432  0.096260
  11  H23    hc     E    9   8   4     1.097   109.662   -59.224  0.096260
  12  H24    hc     E    9   8   4     1.096   111.866    59.968  0.096260
  13  C22    c3     3    8   4   3     1.547   109.323    83.904 -0.442270
  14  H25    hc     E   13   8   4     1.095   111.838   -60.099  0.096260
  15  H26    hc     E   13   8   4     1.097   110.379    59.804  0.096260
  16  H27    hc     E   13   8   4     1.095   110.911   179.524  0.096260
  17  C18    ca     M    8   4   3     1.539   109.379   -35.928  0.041500
  18  C23    ca     B   17   8   4     1.401   122.898  -119.838 -0.308670
  19  C24    ca     S   18  17   8     1.396   121.389  -179.791 -0.071080
  20  H29    ha     E   19  18  17     1.086   120.200  -179.965  0.135000
  21  H28    ha     E   18  17   8     1.084   120.362    -0.006  0.165670
  22  C17    ca     M   17   8   4     1.406   119.990    60.026 -0.308670
  23  H18    ha     E   22  17   8     1.087   119.722    -0.582  0.165670
  24  C16    ca     M   22  17   8     1.392   121.990  -179.978 -0.071080
  25  H17    ha     E   24  22  17     1.084   121.026   179.219  0.135000
  26  C15    ca     M   24  22  17     1.397   119.828    -0.439  0.154650
  27  N1     n      M   26  24  22     1.445   121.410  -178.858 -0.237230
  28  C1     c      B   27  26  24     1.385   116.041   -86.544  0.471270
  29  C25    cc     S   28  27  26     1.494   121.271  -176.435  0.181290
  30  C26    cd     B   29  28  27     1.378   121.071  -169.877 -0.200760
  31  N4     na     B   30  29  28     1.371   105.606  -177.422 -0.262270
  32  C27    cd     B   31  30  29     1.368   107.510     0.333  0.072640
  33  N5     nc     E   32  31  30     1.313   111.349    -0.116 -0.395260
  34  H31    h5     E   32  31  30     1.082   122.610   179.701  0.159280
  35  H33    hn     E   31  30  29     1.010   126.059  -179.477  0.341700
  36  H30    h4     E   30  29  28     1.078   130.888     2.110  0.220940
  37  O1     o      E   28  27  26     1.228   121.133     1.032 -0.567290
  38  C2     c3     M   27  26  24     1.471   118.479    80.006 -0.026980
  39  C10    ca     S   38  27  26     1.522   112.264    55.910 -0.321260
  40  C11    ca     B   39  38  27     1.399   119.539    95.176  0.189540
  41  C12    ca     B   40  39  38     1.394   119.451  -178.845 -0.517240
  42  C13    ca     B   41  40  39     1.395   118.336    -0.216  0.403240
  43  N3     nb     S   42  41  40     1.339   123.505     0.190 -0.688890
  44  C14    ca     S   43  42  41     1.338   117.374     0.148  0.501150
  45  H16    h4     E   44  43  42     1.088   115.802   179.687  0.020300
```

46	H15	h4	E	42	41	40	1.089	120.481	-179.755	0.064920
47	H14	ha	E	41	40	39	1.086	121.143	-179.988	0.201900
48	H13	ha	E	40	39	38	1.088	119.564	0.703	0.086040
49	H1	h1	E	38	27	26	1.093	105.843	171.426	0.050770
50	C3	c	M	38	27	26	1.555	111.431	-76.704	0.887330
51	O2	o	E	50	38	27	1.226	121.164	-23.697	-0.618090
52	N2	n	M	50	38	27	1.361	115.482	161.537	-0.980760
53	H32	hn	E	52	50	38	1.011	118.688	-3.542	0.387480
54	C4	c3	M	52	50	38	1.463	123.049	172.072	0.542290
55	H2	h1	E	54	52	50	1.095	105.400	23.116	-0.005340
56	C5	c3	M	54	52	50	1.536	110.399	141.067	-0.161980
57	H3	hc	E	56	54	52	1.097	109.661	-57.795	0.045720
58	H4	hc	E	56	54	52	1.101	109.077	58.863	0.045720
59	C6	c3	M	56	54	52	1.538	111.446	179.376	-0.037970
60	H5	hc	E	59	56	54	1.099	109.068	65.983	0.020740
61	H6	hc	E	59	56	54	1.097	109.823	-177.644	0.020740
62	C7	c3	M	59	56	54	1.537	111.669	-54.782	-0.026140
63	H7	hc	E	62	59	56	1.098	110.113	177.378	0.014720
64	H8	hc	E	62	59	56	1.100	109.268	-65.957	0.014720
65	C8	c3	M	62	59	56	1.536	111.216	54.798	-0.037970
66	H9	hc	E	65	62	59	1.099	109.330	65.794	0.020740
67	H10	hc	E	65	62	59	1.097	110.396	-177.449	0.020740
68	C9	c3	M	65	62	59	1.537	111.472	-54.978	-0.161980
69	H11	hc	E	68	65	62	1.100	109.610	-65.362	0.045720
70	H12	hc	E	68	65	62	1.095	110.787	176.764	0.045720

LOOP

C15	C24
N5	C25
C14	C10
C9	C4

IMPROPER

C19	C23	C18	C17
C18	C24	C23	H28
C23	C15	C24	H29
C18	C16	C17	H18
C17	C15	C16	H17
C24	C16	C15	N1
C1	C2	N1	C15
C25	N1	C1	O1
C1	C26	C25	N5
C25	H30	C26	N4
C26	C27	N4	H33
H31	N4	C27	N5
C2	C11	C10	C14
C10	C12	C11	H13
C11	C13	C12	H14
C12	H15	C13	N3
C10	H16	C14	N3
C2	N2	C3	O2
C4	C3	N2	H32

DONE
STOP

B.frcmod

remark goes here
MASS

BOND

ANGLE

DIHE
ce-cf-ca-ca 1 0.700 180.000 2.000 same as X -c2-ca-X
ha-cf-ca-ca 1 0.700 180.000 2.000 same as X -c2-ca-X

IMPROPER
ca-ca-ca-oh 1.1 180.0 2.0 Using default value
ca-ca-ca-ha 1.1 180.0 2.0 General improper torsional angle (2 general atom types)
c3-o -c -os 10.5 180.0 2.0 General improper torsional angle (2 general atom types)
ce-o -c -os 10.5 180.0 2.0 General improper torsional angle (2 general atom types)
c -cf-ce-ha 1.1 180.0 2.0 Using default value
ca-ce-cf-ha 1.1 180.0 2.0 Using default value
ca-ca-ca-cf 1.1 180.0 2.0 Using default value

NONBON

B.prep

```
 0 0 2

This is a remark line
molecule.res
LIG INT 0
CORRECT OMIT DU BEG
 0.0000
 1 DUMM DU M 0 -1 -2 0.000 .0 .0 .00000
 2 DUMM DU M 1 0 -1 1.449 .0 .0 .00000
 3 DUMM DU M 2 1 0 1.523 111.21 .0 .00000
 4 O5 oh M 3 2 1 1.540 111.208 -180.000 -0.661420
 5 H13 ho E 4 3 2 0.969 32.501 -71.635 0.472560
 6 C9 ca M 4 3 2 1.380 106.335 29.256 0.229310
 7 C8 ca B 6 4 3 1.389 124.904 -31.437 -0.338840
 8 C5 ca S 7 6 4 1.399 119.849 179.651 -0.169490
 9 H5 ha E 8 7 6 1.086 119.417 -179.765 0.173080
 10 H6 ha E 7 6 4 1.089 119.771 -0.002 0.182820
 11 C7 ca M 6 4 3 1.406 114.944 148.158 0.382290
 12 O3 oh S 11 6 4 1.364 120.336 0.047 -0.631110
 13 H11 ho E 12 11 6 0.973 107.358 1.021 0.467930
 14 C4 ca M 11 6 4 1.390 119.613 -179.631 -0.355470
 15 H4 ha E 14 11 6 1.086 118.417 -179.536 0.226710
 16 C3 ca M 14 11 6 1.403 120.822 -0.176 -0.007670
 17 C1 c3 M 16 14 11 1.515 120.614 -179.407 -0.026000
 18 H1 hc E 17 16 14 1.096 110.404 -40.563 0.053830
 19 H2 hc E 17 16 14 1.095 109.955 -158.518 0.053830
 20 C2 c3 M 17 16 14 1.541 115.272 80.463 0.066660
 21 C6 c B 20 17 16 1.531 112.925 55.465 0.807380
 22 O2 os S 21 20 17 1.353 109.686 63.150 -0.424070
 23 C19 c3 3 22 21 20 1.438 115.086 179.479 -0.158960
 24 H14 h1 E 23 22 21 1.094 110.426 61.005 0.123560
 25 H15 h1 E 23 22 21 1.093 110.414 -59.625 0.123560
 26 H16 h1 E 23 22 21 1.090 105.584 -179.253 0.123560
 27 O4 o E 21 20 17 1.206 125.958 -116.973 -0.558180
 28 H3 h1 E 20 17 16 1.095 109.519 175.068 0.075070
 29 O1 os M 20 17 16 1.426 108.308 -65.794 -0.441140
 30 C10 c M 29 20 17 1.367 116.025 -159.191 0.888370
 31 O6 o E 30 29 20 1.216 122.967 4.093 -0.606440
 32 C12 ce M 30 29 20 1.470 110.405 -177.056 -0.527690
 33 H7 ha E 32 30 29 1.084 116.784 1.946 0.203150
 34 C13 cf M 32 30 29 1.349 119.878 -178.600 0.035410
 35 H8 ha E 34 32 30 1.089 116.001 0.192 0.123040
 36 C11 ca M 34 32 30 1.457 128.022 -179.729 0.126660
 37 C14 ca M 36 34 32 1.410 118.430 -179.708 -0.399590
 38 H9 ha E 37 36 34 1.089 119.469 -0.072 0.203820
 39 C16 ca M 37 36 34 1.386 120.915 179.949 0.230250
 40 O7 oh S 39 37 36 1.378 124.842 179.995 -0.658900
 41 H17 ho E 40 39 37 0.968 109.803 -0.663 0.476040
 42 C18 ca M 39 37 36 1.406 120.399 -0.013 0.390820
 43 O8 oh S 42 39 37 1.358 120.511 -179.989 -0.618420
 44 H18 ho E 43 42 39 0.972 107.586 -0.186 0.468830
 45 C17 ca M 42 39 37 1.396 119.241 0.021 -0.327200
```

```
 46  H12  ha   E  45  42  39   1.085  118.422  179.969  0.220410
 47  C15  ca   M  45  42  39   1.390  120.320   -0.046 -0.210310
 48  H10  ha   E  47  45  42   1.085  119.052  179.864  0.191970


LOOP
   C3   C5
  C15  C11

IMPROPER
   C7   C8   C9   O5
   C9   C5   C8   H6
   C3   C8   C5   H5
   C4   C9   C7   O3
   C7   C3   C4   H4
   C1   C4   C3   C5
   C2   O4   C6   O2
  C12   O6  C10   O1
  C10  C13  C12   H7
  C11  C12  C13   H8
  C14  C15  C11  C13
  C11  C16  C14   H9
  C14  C18  C16   O7
  C16  C17  C18   O8
  C18  C15  C17  H12
  C11  C17  C15  H10

DONE
STOP
```

C.frcmod

remark goes here
MASS

BOND

ANGLE

DIHE

IMPROPER
ca-o -no-o 1.1 180.0 2.0 Using default value
ca-ca-ca-no 1.1 180.0 2.0 Using default value
ca-ca-ca-ha 1.1 180.0 2.0 General improper torsional angle (2 general atom types)
ca-ca-ca-n 1.1 180.0 2.0 Using default value
c -ca-n -hn 1.1 180.0 2.0 General improper torsional angle (2 general atom types)
ca-n -c -o 10.5 180.0 2.0 General improper torsional angle (2 general atom types)
c -ca-ca-ca 1.1 180.0 2.0 Using default value
ca-ca-ca-oh 1.1 180.0 2.0 Using default value

NONBON

C.prep

```
    0    0    2

This is a remark line
molecule.res
LIG    INT   0
CORRECT       OMIT DU    BEG
  0.0000
   1  DUMM   DU     M     0  -1  -2      0.000       .0         .0        .00000
   2  DUMM   DU     M     1   0  -1      1.449       .0         .0        .00000
   3  DUMM   DU     M     2   1   0      1.523    111.21        .0        .00000
   4  O3     o      M     3   2   1      1.540    111.208  -180.000 -0.442850
   5  N2     no     M     4   3   2      1.232     47.501     0.000  0.711610
   6  O4     o      E     5   4   3      1.233    124.660   180.000 -0.442850
   7  C7     ca     M     5   4   3      1.464    117.618    -0.000 -0.010400
   8  C10    ca     B     7   5   4      1.392    119.826     0.000 -0.118590
   9  C5     ca     S     8   7   5      1.388    119.722   180.000 -0.344720
  10  H2     ha     E     9   8   7      1.080    120.795   180.000  0.232230
  11  H5     ha     E     8   7   5      1.083    119.494     0.000  0.196860
  12  C9     ca     M     7   5   4      1.393    118.781   180.000 -0.076980
  13  H4     ha     E    12   7   5      1.082    120.181    -0.000  0.187660
  14  C4     ca     M    12   7   5      1.384    118.411   180.000 -0.163450
  15  Cl1    cl     E    14  12   7      1.765    118.027   180.000 -0.068020
  16  C1     ca     M    14  12   7      1.417    122.113     0.000  0.486820
  17  N1     n      M    16  14  12      1.392    118.462   180.000 -0.601030
  18  H1     hn     E    17  16  14      1.009    113.680    -0.000  0.259990
  19  C3     c      M    17  16  14      1.395    128.221   180.000  0.755950
  20  O2     o      E    19  17  16      1.220    121.892     0.000 -0.526140
  21  C2     ca     M    19  17  16      1.506    115.441   180.000 -0.273480
  22  C8     ca     S    21  19  17      1.408    122.590    -0.000 -0.021530
  23  H3     ha     E    22  21  19      1.083    121.575    -0.000  0.164200
  24  C6     ca     M    21  19  17      1.420    119.958   180.000  0.495680
  25  O1     oh     S    24  21  19      1.353    120.253     0.000 -0.604380
  26  H8     ho     E    25  24  21      0.971    108.433   180.000  0.451210
  27  C11    ca     M    24  21  19      1.403    119.708   180.000 -0.446320
  28  H6     ha     E    27  24  21      1.089    118.858   180.000  0.190230
  29  C13    ca     M    27  24  21      1.387    121.748    -0.000  0.087130
  30  H7     ha     E    29  27  24      1.085    120.689   180.000  0.141440
  31  C12    ca     M    29  27  24      1.393    118.809    -0.000 -0.127610
  32  Cl2    cl     M    31  29  27      1.756    119.921   180.000 -0.092640


LOOP
   C1    C5
  C12    C8

IMPROPER
   C7    O3    N2    O4
   C9   C10    C7    N2
   C7    C5   C10    H5
   C1   C10    C5    H2
   C4    C7    C9    H4
   C9    C1    C4   Cl1
```

```
  C4   C5   C1   N1
  C3   C1   N1   H1
  C2   N1   C3   O2
  C3   C8   C2   C6
  C2  C12   C8   H3
  C2  C11   C6   O1
  C6  C13  C11   H6
 C11  C12  C13   H7
  C8  C13  C12  Cl2

DONE
STOP
```

D.frcmod

```
remark goes here
MASS

BOND

ANGLE

DIHE
ca-ca-cc-cd   1   0.700   180.000   2.000   same as X -c2-ca-X
ca-ca-cc-c    1   0.700   180.000   2.000   same as X -c2-ca-X
cc-cd-os-ca   1   1.050   180.000   2.000   same as X -c2-os-X
h4-cd-os-ca   1   1.050   180.000   2.000   same as X -c2-os-X

IMPROPER
c2-c3-c2-c3    1.1   180.0   2.0   Using default value
c2-c3-c2-ha    1.1   180.0   2.0   Using default value
ca-ca-ca-oh    1.1   180.0   2.0   Using default value
ca-ca-ca-ha    1.1   180.0   2.0   General improper torsional angle (2 general atom types)
ca-ca-ca-cc    1.1   180.0   2.0   Using default value
c -ca-cc-cd    1.1   180.0   2.0   Using default value
cc-h4-cd-os    1.1   180.0   2.0   Using default value
ca-cc-c -o    10.5   180.0   2.0   General improper torsional angle (2 general atom types)
c -ca-ca-ca    1.1   180.0   2.0   Using default value
ca-ca-ca-os    1.1   180.0   2.0   Using default value

NONBON
```

D.prep

```
    0    0    2

This is a remark line
molecule.res
LIG     INT   0
CORRECT       OMIT DU    BEG
  0.0000
   1  DUMM   DU    M    0  -1  -2      0.000       .0          .0        .00000
   2  DUMM   DU    M    1   0  -1      1.449       .0          .0        .00000
   3  DUMM   DU    M    2   1   0      1.523    111.21         .0        .00000
   4  C19    c3    M    3   2   1      1.540    111.208   -180.000 -0.442100
   5  H9     hc    E    4   3   2      1.094    116.833     79.438  0.121810
   6  H10    hc    E    4   3   2      1.099     10.259    113.814  0.121810
   7  H11    hc    E    4   3   2      1.099    107.197   -159.252  0.121810
   8  C16    c2    M    4   3   2      1.511    101.955    -42.834  0.337910
   9  C20    c3    3    8   4   3      1.512    114.399    -54.449 -0.442100
  10  H12    hc    E    9   8   4      1.092    113.523    174.541  0.121810
  11  H13    hc    E    9   8   4      1.098    110.177    -64.516  0.121810
  12  H14    hc    E    9   8   4      1.099    110.791     52.853  0.121810
  13  C12    c2    M    8   4   3      1.347    120.376    127.607 -0.406140
  14  H5     ha    E   13   8   4      1.088    118.268     -0.431  0.200370
  15  C4     c3    M   13   8   4      1.514    128.320    178.596 -0.053590
  16  H1     hc    E   15  13   8      1.097    110.805      4.975  0.094560
  17  H2     hc    E   15  13   8      1.094    108.820   -111.484  0.094560
  18  C2     ca    M   15  13   8      1.521    112.753    126.966 -0.088300
  19  C7     ca    B   18  15  13      1.398    118.084    -62.924  0.178540
  20  O2     oh    S   19  18  15      1.381    123.042      2.220 -0.566810
  21  H15    ho    E   20  19  18      0.977    108.258     33.060  0.369990
  22  C11    ca    B   19  18  15      1.404    122.107   -175.742  0.351600
  23  O4     oh    S   22  19  18      1.360    119.804    179.786 -0.595850
  24  H16    ho    E   23  22  19      0.974    106.274      2.406  0.433580
  25  C13    ca    B   22  19  18      1.391    119.363     -0.321 -0.345870
  26  C8     ca    S   25  22  19      1.394    119.253     -1.133 -0.144400
  27  H3     ha    E   26  25  22      1.085    119.274   -177.523  0.145020
  28  H6     ha    E   25  22  19      1.085    119.241   -179.533  0.217710
  29  C1     ca    M   18  15  13      1.414    123.742    119.533 -0.083590
  30  C3     cc    M   29  18  15      1.488    122.306     -3.967 -0.241960
  31  C10    cd    B   30  29  18      1.356    119.893    122.316  0.185310
  32  O1     os    E   31  30  29      1.351    125.794    175.209 -0.346430
  33  H4     h4    E   31  30  29      1.084    123.364     -4.675  0.153410
  34  C6     c     M   30  29  18      1.470    121.911    -62.493  0.807910
  35  O3     o     E   34  30  29      1.252    122.497      4.747 -0.609140
  36  C5     ca    M   34  30  29      1.453    115.670   -175.014 -0.657450
  37  C9     ca    M   36  34  30      1.409    121.093     -0.117  0.572420
  38  C15    ca    M   37  36  34      1.387    123.161    179.941 -0.618860
  39  H7     ha    E   38  37  36      1.083    121.312    179.987  0.256940
  40  C18    ca    M   38  37  36      1.399    117.608     -0.188  0.629970
  41  O6     oh    S   40  38  37      1.359    116.654   -179.960 -0.601230
  42  H18    ho    E   41  40  38      0.970    109.206    179.867  0.437430
  43  C17    ca    M   40  38  37      1.401    121.681      0.089 -0.705610
  44  H8     ha    E   43  40  38      1.086    121.309    179.995  0.255050
  45  C14    ca    M   43  40  38      1.394    119.894     -0.085  0.660370
```

```
 46  O5    oh   M   45  43  40    1.339   119.249  -179.977 -0.609620
 47  H17   ho   E   46  45  43    1.000   106.245   179.808  0.445500


LOOP
   C1    C8
   C9    O1
  C14    C5

IMPROPER
  C12   C19   C16   C20
  C16    C4   C12    H5
   C4    C7    C2    C1
   C2   C11    C7    O2
   C7   C13   C11    O4
  C11    C8   C13    H6
  C13    C1    C8    H3
   C2    C8    C1    C3
   C6    C1    C3   C10
   C3    H4   C10    O1
   C5    C3    C6    O3
   C6    C9    C5   C14
   C5   C15    C9    O1
   C9   C18   C15    H7
  C15   C17   C18    O6
  C18   C14   C17    H8
   C5   C17   C14    O5

DONE
STOP
```
